# Feasibility and effectiveness of tailored interventions for two populations at high-risk of malaria in Senegal: Koranic school children and gold miners

**DOI:** 10.1371/journal.pgph.0004569

**Published:** 2025-04-29

**Authors:** Sarah Gallalee, Demba Kande, Tidiane Thiam, Henry Ntuku, Caterina Guinovart, Laura Merriman, Abiboulaye Sall, Moustapha Cissé, Aichatou Barry Diouf, Mamadou Diop, Baba Camara, Niene Seck, Faith De Amaral, Roly Gosling, Bryan Greenhouse, Yakou Dieye, Jennifer Smith, Adam Bennett

**Affiliations:** 1 Malaria Elimination Initiative, Institute for Global Health Sciences, University of California San Francisco, San Francisco, California, United States of America; 2 PATH Malaria Control and Elimination Partnership in Africa, Dakar, Senegal; 3 PATH Malaria Control and Elimination Partnership in Africa, Seattle, Washington, United States of America; 4 Barcelona Institute for Global Health, Hospital Clínic - Universitat de Barcelona, Barcelona, Spain; 5 Ministère de la Santé et de l’Action Sociale, Dakar, Senegal; 6 Division of HIV, Infectious Diseases, and Global Medicine, Zuckerberg San Francisco General Hospital, University of California San Francisco, San Francisco, California, United States of America; 7 Department of Disease Control, London School of Hygiene and Tropical Medicine, London, United Kingdom; 8 Department of Epidemiology and Biostatistics, University of California San Francisco, San Francisco, California, United States of America; Tulane University School of Public Health and Tropical Medicine, UNITED STATES OF AMERICA

## Abstract

Senegal has made significant progress in reducing the malaria burden over the last decade. However, malaria remains a major cause of morbidity in some regions and key challenges exist among high-risk populations who have high exposure to mosquitos, but low coverage and use of vector control measures and limited access to healthcare. Two identified high-risk populations are goldminers and talibés (Koranic school students). We conducted a controlled pre/post survey to measure the impact of targeted malaria interventions, including expansion of active community case management and distribution of LLINs, on reported LLIN usage and *Plasmodium falciparum* infection prevalence at mining sites and Koranic schools (daaras) during the high transmission season in Senegal. We randomly assigned four health facility catchment areas in Kaolack (a city with many daaras) and four in Saraya (a district with gold mining sites) to intervention or control groups. Surveys were conducted pre (Oct 2021; n = 1740 talibés and gold miners) and post (Feb 2022; n = 2200) delivery of the intervention package to assess intervention coverage and infection prevalence by rapid diagnostic test and qPCR. We compared infection prevalence and self-reported LLIN usage, by group and arm, between the two time periods using a difference in difference framework with binomial generalized linear mixed models. Among the talibés, the package of interventions was associated with an adjusted 12.6-percentage point relative reduction in RDT-derived malaria prevalence (p < 0.05, adjusted risk difference: -12.6, 95% CI: -2.7, -22.4) and an adjusted 44.0-percentage point increase in reported prior night net use (p < 0.001, aRD: 44.0, 95% CI: 36.3, 51.6) in the intervention group compared to the counterfactual. However, among the gold miners there was no measured association between the package of interventions and these outcomes. While there was high acceptability in both groups, interventions should be tailored to address high mobility amongst gold miners and maximize impact.

## Introduction

The successful implementation of malaria control interventions in Senegal including distribution of long-lasting insecticide treated nets (LLINs), indoor residual spraying (IRS), seasonal malaria chemoprevention, and improved case management led to a decrease in malaria incidence (from 61 to 42 per 1,000 population) and incidence of malaria deaths (from 5.7 to 0.27 per 1,000 population) between 2010 to 2019 [1]. Since 2019, progress has stalled, with malaria incidence increasing slightly [1,2]. The changing epidemiologic context during the last decade has led to more heterogeneous transmission across Senegal in which specific high-risk populations (HRPs) are at greater risk of malaria infection due to shared characteristics such as demographics, behavior, occupation, or geographic location [3,4]. Anecdotal reports, routine malaria surveillance data, a formative assessment, and a case-control study informed the identification of Koranic boarding school students (talibés) in Kaolack district and gold miners in Saraya district as populations potentially at higher risk of malaria infection in Senegal [5].

Talibés are mainly boys aged five to eighteen years old who live in traditional Koranic boarding schools (“daaras”) under the responsibility of a school master. Talibés often live in poor housing conditions, have less access to malaria control tools and health care, and are exposed to mosquitoes while outside in the evenings begging [5]. Other studies in Senegal have found that adolescents are particularly at risk of infection and vulnerable to malaria [6,7] and research in other African countries indicates that while school age children and adolescents experience high burdens of malaria, they are often less likely to be prioritized for interventions than other groups [8,9]. Gold miners are considered a malaria high-risk population globally; despite regional variations in climate, vectors, and populations, malaria is associated with mining operations in three separate continents [10]. Recent studies have found that gold miners are a high-risk population in Senegal that require targeted interventions [5]. Gold miners in Senegal include citizens of Senegal and neighboring countries, are highly mobile, have low access to healthcare personnel, are often missed by routine interventions such as IRS due to the remoteness of worksites or because they sleep in makeshift housing that is not sprayable, and are exposed to mosquitoes due to the poor housing conditions and while socializing outside at night [5].

While there is evidence that talibés and gold miners are at higher risk of malaria, there has been limited research on targeting interventions to reach these populations. Both groups are highly exposed to mosquitoes and are more difficult to include in routine interventions due to congregate living away from households. This study evaluated the implementation of a package of interventions targeted to talibés and gold miners in Senegal including enhanced community case management, targeted delivery of long-lasting insecticide treated nets (LLINs), and malaria education.

## Materials and methods

### Study area

**Fig 1:**
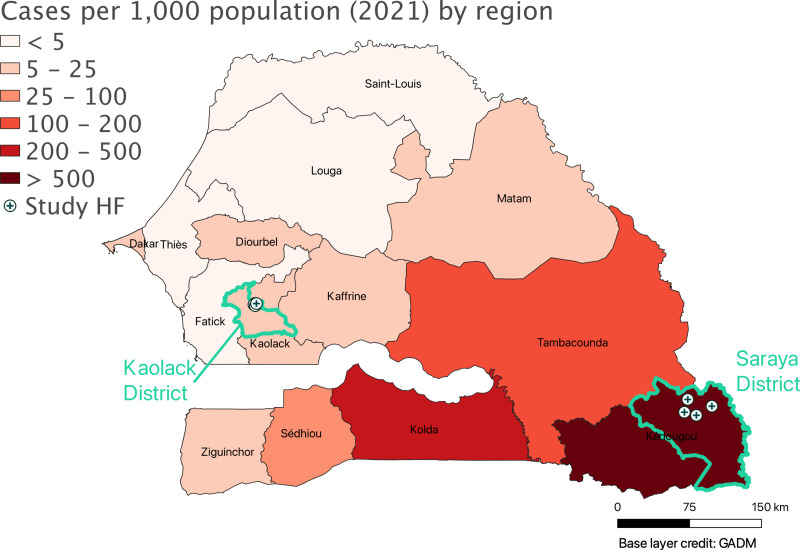
2021 malaria incidence by region in Senegal and locations the study districts and health facilities. HF health facility. Base layer credit: GADM [13].

This study took place in Kaolack district, which has the largest number of daaras and talibés in Senegal and Saraya district, where gold miners are active (Fig 1) [5]. Malaria is highly seasonal in both districts, with the typical high transmission season running from July to December (during and just after the rainy season). Malaria is almost entirely due to *Plasmodium falciparum* [11]. In 2020, Kaolack reported an annual incidence of 9 cases per 1,000 population and Saraya reported 627 cases per 1,000 population and [12].

The study sites included the catchment areas of four health facilities in each district: Khossanto, Diakhaling, Mamakhono and Sambrambougou in the largely rural Saraya health district, and Nimzatt, Ngane, Thioffac and Tabangoye in a largely urban area of Kaolack health district. The total recorded population within the catchment areas of these health facilities was approximately 14,000 and 81,000 in Saraya and Kaolack, respectively. Health facilities were selected based on the known presence of talibés or gold miners in their catchments and historically high malaria incidence. An enumeration was conducted prior to the start of the study to estimate the number of daaras, talibés, mining sites, and gold miners in the study catchments; the managers of the daaras and the mines provided estimates at each site. The enumerated targeted population was 5,762 gold miners at gold mines in Saraya and 4,118 talibés in daaras in Kaolack.

### Study design

This study was a controlled pre- and post-intervention evaluation over one high transmission season. The baseline survey took place in October 2021; the endline in January through March 2022. Two health facility catchments in each district were randomly assigned to receive the intervention and two in each district were assigned to be control. Randomization was conducted at the health facility catchment level because activities are implemented by the Programme National de Lutte Contre le Paludisme (PNLP) at this level, the intervention was implemented by the PNLP using their routine structure, and it was not operationally feasible to randomize at the site level (daara or gold mine).

All facilities received routine malaria control activities, including case management with rapid diagnostic tests (RDTs) and artemisinin-based combination therapy (ACT) with artemether-lumefantrine in Kaolack and artesunate-amodiaquine in Saraya in health facilities and by community health workers (DSDOMs), distribution of LLINs through universal coverage mass campaigns (the last campaign prior to the study was in 2019) and through antenatal care (ANC), seasonal malaria chemoprevention (SMC) targeting children aged 3 months to 10 years, and indoor residual spraying (IRS) of insecticide to sleeping structures within some villages.

### Intervention

The intervention group received a three-part intervention package in addition to routine malaria control. The first item in the package was enhanced community case management in which a peer community health worker was established at each of the targeted daaras (DSDAARA) or gold mining sites (DSDOR). The daara school master or owner of the gold mine was asked to nominate potential candidates of at least eighteen years of age, who were then assessed on criteria determined during the formative assessment including availability, responsibility, knowledge of the site, and interpersonal skills. One of the talibés or miners was selected, trained, and provided with necessary supplies for diagnosis and treatment of uncomplicated malaria. These community health workers were also trained to conduct weekly visits to all peers at their site to test for malaria in those with fever, history of fever in the last forty-eight hours, or symptoms suggestive of malaria (PECADOM+). The second item in the package of interventions was targeted delivery of LLINs; LLINs were distributed to gold miners (one net per one miner) and the heads of the daaras (one net per two talibés) during the baseline cross-sectional survey (October 2021) and via DSDAARA or DSDOR to any new students or gold miners who arrived after the baseline survey. The third item in the package of interventions was malaria education sessions that were conducted by the DSDAARA or DSDOR throughout the study period on causes, symptoms, and prevention of malaria, the importance of seeking care rapidly after the onset of symptoms, and demonstrations on how to use and hang the LLINs.

### Outcomes

The two primary outcomes evaluated in this study were prevalence of *Plasmodium falciparum* infection using RDTs (SD BIOLINE Malaria Ag P.f/pan, Standard Diagnostics, Korea) and polymerase chain reaction (PCR), and self-reported LLIN coverage defined as the proportion of individuals who reported sleeping under an LLIN the previous night.

The secondary outcome evaluated included incidence of passively detected malaria cases at the health facility catchment level (confirmed by RDT or microscopy at the health facility and by community health workers) among the target populations and in the general population to determine if there was evidence of a change in malaria incidence in the intervention health facility catchments compared to the counterfactual. We also evaluated the operational feasibility and acceptability of the malaria interventions via DSDAARA, daara school master, DSDOR, and gold miner reported indicators on LLIN coverage, malaria testing and treating (and PECADOM+ among gold miners), and malaria knowledge (to assess the education component). Acceptability was assessed via self-reported indicators on how comfortable the DSDOR and DSDAARA were with performing the interventions and how comfortable the talibés and gold miners were with receiving the interventions from their peers.

### Sample size and sampling

Sample size calculations for the primary outcomes were powered to detect at least a 10% absolute pre-post difference in intervention coverage (LLINs) in target populations between control and intervention groups in each district, assuming 50% coverage in control areas with 80% power at the 5% significance level and allowing for 10% non-response [14]. This sample size provided 80% power to detect an absolute reduction of 6% in malaria infection prevalence in gold miners between control and intervention groups, assuming 15% prevalence in the control group, and a 5% significance level. In talibés, the sample size provided 80% power to detect an absolute reduction of 5% in the prevalence of malaria between control and intervention groups, assuming a prevalence of 10% in the control group, and a 5% significance level. A sample of 1,740 individuals (435 individuals per arm per district) was planned for the baseline survey. After the baseline survey was conducted, the design effect calculated was 1.25; this was adjusted for in the endline survey; the sample size was increased to 550 per arm per district, with a total of 2,200 individuals in the two districts. During the endline survey, efforts were made to find and sample participants that were also present at baseline, as much as possible.

For the surveys of talibés, sites (daaras) were selected with probability proportional to size of the daaras, and the number of students to sample at each daara was fixed at twenty-two [15]. For the gold miners, all gold mining sites in operation in the study area at the time of the survey were included. Within each mine, the number of gold miners recruited was proportional to the size of the site. At each site at baseline, participants were selected using a random number table. At endline, the same participants recruited for the baseline survey were invited to participate again in the endline survey, when possible. Additional participants were selected via a random number table and invited to participate until the sample size was reached.

### Data collection

The baseline survey occurred in October 2021; the start date of recruitment was October 6^th^, 2021. The endline survey was conducted between January and March 2022; the end date of recruitment was March 9^th^, 2022. Talibés were eligible for inclusion in the survey if at the time of the intervention they were a boarding learner at a selected daara within the four health facility catchment areas in Kaolack District. Gold miners were eligible if at the time of the intervention they were working as a gold miner at a selected gold mining site within the four health facility catchment areas in Saraya District.

A standardized survey instrument was developed to collect information about the gold mining site or Daara where participants work/stay, demographic characteristics, occupation, coverage of individual and household level malaria preventive measures (including IRS, LLINs, SMC), and behavioral risk factors. For the talibés, some survey questions were asked of the talibés directly, and some about the daaras were asked of the school masters (e.g., size of the daara, number of bed nets, and questions on assets such as electricity). The DSDOR, DSDAARA, and school masters were also interviewed at endline on questions regarding the feasibility and acceptability of the peer community health worker intervention. This included an additional indicator on LLIN coverage; for gold miners coverage was defined as one net per one gold miner, for talibés, we assessed daara-level LLIN coverage as the proportion of daaras that had a least one LLIN per two talibés [16,17]. Blood was collected by fingerprick from all participants for immediate malaria testing by RDT and dried blood spots (DBS) on filter paper.

For the secondary outcome of passively detected malaria incidence at the health facility level, routinely collected monthly surveillance data of RDT or microscopy-confirmed passively detected cases were collected, disaggregated by HRP status (gold miner or talibé) from July 2021- September 2021 (pre-intervention) and during/post intervention (October 2021-January 2022), with the week of the intervention start considered a washout period.

### Data processing

Data entry was carried out using electronic questionnaires programmed in Open Data Kit (ODK [18]) on tablet devices and entries were uploaded to secure cloud-based databases. Data management, cleaning, and analysis were conducted in STATA 16.1, R 4.1.2, and QGIS 3.12.

To represent socio-economic status (SES), we conducted a principal component analysis (PCA) of binary assets (e.g. electricity, radio, etc) and categorical variables of water source, toilet facilities, and fuel/energy for cooking [19]. For the talibés, PCA was categorized into quartiles; for gold miners the quartiles were condensed into two groups (below and above 50^th^ percentile) during analysis to avoid small cells. Estimates of average monthly precipitation (rainfall in mm) were obtained from CHIRPS at 1 km spatial resolution [20]. Monthly enhanced vegetation index (EVI) and monthly average land surface temperature (LST) were obtained from MODIS at 1km spatial resolution using the MODIStsp package in R [21–23]. Averages of precipitation, EVI, and LST were calculated with a two-month lag leading up to the baseline and endline surveys and extracted to point for the individual study sites. Euclidean distance from each site to the closest health facility was calculated in QGIS and grouped into four categories (for gold miners the four groups were condensed into three groups during analysis to avoid small cells).

DBS were assayed by quantitative polymerase chain reaction (qPCR) at the University of California, San Francisco. DBS were placed in desiccant and stored at 4°C. DNA was extracted using the chelex-based extraction method [24,25] with a final volume of 100uL-150uL, then stored at -20°C. Following extraction, qPCR was conducted to quantify parasitemia of *Plasmodium falciparum* using var gene acidic terminal sequence (varATS) assay [26]. Samples were considered positive for *Plasmodium falciparum* if the parasite quantity was greater than 1p/uL.

### Data analysis

The talibés and gold miners were analyzed as separate groups. For the summary analysis of talibés, basic sampling probability weights were computed; weighted proportions are presented for categorical variables, and weighted means (with standard deviation) are presented for continuous variables (unless otherwise stated) [27]. For the gold miners, unweighted proportions and means are presented because the sample is self-weighted.

The main outcomes of malaria prevalence (RDT-derived and qPCR-derived) and self-reported LLIN coverage were analyzed using a difference in difference (DID) approach with multivariable binomial generalized linear mixed models with a logit link. The models accounted for clustered standard errors at the site (daara or gold mine) and the adjusted models included covariates relevant to each population. Covariates were determined *a priori* and included age group, number of people living in the daara or mine, socioeconomic status, education level (for miners only), distance to nearest health facility, seasonal malaria chemoprevention (for talibés only), indoor residual spraying (for miners only), type of living/sleeping structure (for miners only) and environmental factors. Covariates were assessed for collinearity; if two variables were collinear, the most interpretable or programmatically useful was kept. Using marginal means, the risk difference (RD; percentage point change) was interpreted to estimate the absolute intervention effect between the intervention group and the counterfactual. Odds ratios for the interaction term of pre-post * intervention-control are also presented, representing the relative effect of the intervention.

There was a loss in the field of DBS samples, primarily from one control health facility catchment (Thioffac) in Kaolack at endline. Therefore, the analysis of malaria prevalence with qPCR among the talibés was restricted to include only daaras that had DBS available at both baseline and endline (a single control group). Among the gold miners, a sensitivity analysis restricted to individuals who reported at endline that they were also present at baseline was conducted to remove bias due to population turnover.

For the secondary outcome of passive malaria case incidence at the health facility level, the approach was DID with a negative binomial regression model, comparing averages across the pre- and post-intervention periods, with fixed effects at the health facility, population as the offset, and controlling for environmental covariates (averages of precipitation, EVI, and LST with a two-month lag). Models were run using the general population (population data from the census) in the health facility catchments of the two districts and subgroup analyses for each HRP group (population data from the study enumeration). Using marginal means, the risk difference (difference in number of cases per 1,000 population) was interpreted to estimate intervention effect between the intervention group and the counterfactual.

### Ethics statement

Institutional Review Board (IRB) approval for this study was obtained from The Senegalese *Comité National d’Éthique pour la Recherche en Santé* (CNERS) and the WCG IRB. An acknowledgement of reliance on the WCG IRB was obtained from the University of California, San Francisco IRB. Informed consent (available in English and translated to French) was read to each participant. Written informed consent was obtained from all participants or from a parent or guardian; consent was documented via participant signature on the informed consent form and witnessed by research staff. For individuals 18 years of age or younger, informed consent was obtained from a parent or guardian. For individuals ages 12–18, written informed consent was obtained from the minor in addition to the consent of the parent or guardian. Some of the findings in this paper have been drawn from a dissertation chapter from 2023 [28].

## Results

**Fig 2:**
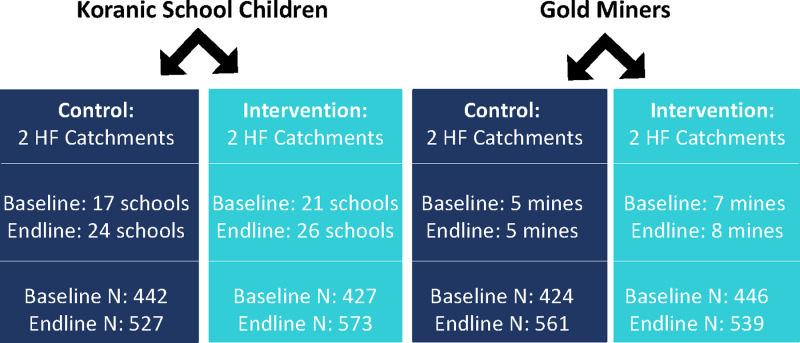
Diagram of randomization at the health facility level and number of sites and participants in each group. HF health facility, N number of participants.

The total number of participants in the evaluation included 1,739 individuals at baseline and 2,200 at endline. Fig 2 depicts the number of sites (daaras or gold mines) in the intervention and control groups during the pre- and post- intervention surveys.

### Talibés

#### Descriptive analysis.

Table 1 presents the characteristics of the talibés and daaras by group. The talibés were mainly male (95.8%) and aged 10–15 years. The median number of people living in the daaras was 48 (ranging from a minimum of 15 to a maximum of 325). At endline, 300 talibés (56.9%) in the control group and 386 talibés (67.4%) in the intervention group reported they were present at baseline.

**Table 1:** Talibés: participant characteristics, malaria prevalence, and LLIN usage.

	Baseline Control	Baseline Intervention	Endline Control	Endline Intervention
	N = 442 (%*, 95% CI)	N = 427 (%*, 95% CI)	N = 527 (%*, 95% CI)	N = 573 (%*, 95% CI)
**Health facility**				
Ngane	136 (29.4, 18.8-42.8)	—	123 (20.8, 13.1-31.4)	—
Nimzatt	—	231 (57.1, 44.9-68.5)	—	310 (53.8, 43.1-64.2)
Taba Ngoye	—	196 (42.9, 31.5-55.0)	—	263 (46.2, 35.8-56.9)
Thioffac	306 (70.5, 57.1-81.2)	—	404 (79.2, 68.6-86.9)	—
**Age group (years)**				
5-9	168 (38.3, 32.0-44.9)	131 (31.5, 25.6-38.0)	173 (34.1, 19.8-38.6)	146 (25.1, 21.2-29.4)
10-15	242 (55.1, 49.2-60.9)	236 (52.6, 46.1-59.0)	328 (61.2, 56.6-65.6)	355 (62, 56.7-66.3)
16 and older	32 (6.6, 4.2-10.4)	60 (15.9, 10.9-22.8)	26 (4.7, 3.0-7.3)	72 (13.3, 9.8-17.8)
**Sex**				
Female	33 (6.7, 3.7-11.9)	5 (2.9, 0.9-8.6)	36 (6.7, 4.1-11.0)	8 (1.6, 0.8-3.6)
Male	409 (93.3, 88.1-96.3)	422 (97.1, 91.4-99.1)	491 (93.3, 89.0-95.9)	565 (98.4, 96.4-99.2)
**# of people in Daara, median (IQR, CI)**	51 (36-65, 46.1-55.9)	40 (36-66, 30.7-49.3)	56 (36.5-72.5, 46.7-65.3)	47.5 (34-67, 45.5-49.5)
**SES (PCA Quartile)**				
0-25.1	142 (35.3, 23.7-48.9)	83 (19.0, 11.3-30.4)	114 (20.8, 13.1-31.4)	141 (23.1, 15.3-33.2)
25.1-50	28 (5.9, 2.0-16.0)	108 (23.8, 14.9-35.8)	99 (20.8, 13.1-31.5)	218 (38.5, 28.7-49.3)
50.1-75	84 (17.6, 9.6-30.2)	161 (38.1, 27.2-50.4)	292 (54.2, 42.9-65.0)	130 (23.1, 15.3-33.3)
75.1-100	188 (41.2, 28.9-54.7)	75 (19.0, 11.3-30.4)	22 (4.2, 1.4-11.6)	84 (15.4, 9.1-24.8)
**Treated with SMC**				
No	28 (6.0, 3.5-10.1)	42 (9.9, 6.5-14.7)	109 (21.0, 17.3-25.2)	34 (6.4, 4.1-9.7)
Ineligible/No response	223 (49.1, 43.0-55.2)	246 (57.4, 50.9-63.6)	254 (46.5, 41.3-51.7)	371 (65.2, 60.3-69.9)
Yes	191 (44.9, 38.3-51.7)	139 (32.8, 26.7-39.5)	164 (32.6, 27.6-38.0)	168 (28.4, 23.9-33.4)
**EVI, mean (SD, CI)**	0.26 (0.07, 0.24-0.27)	0.21 (0.08, 0.19-0.23)	0.21 (0.03, 0.20-0.21)	0.18 (0.04, 0.18-19)
**Temp. (C), mean (SD, CI)**	29.8 (0.0, 29.8-29.8)	29.4 (0.4, 29.3-29.5)	35.2 (0.0, 35.2-35.2)	35.5 (1.3, 35.2-35.8)
**Precip. (mm), mean (SD, CI)**	214.8 (0.0, 214.8-214.8)	216.9 (9.2, 214.6-219.2)	1.3 (0.0, 1.3-1.3)	1.3 (0.0, 1.3-1.3)
**Distance to nearest HF (km)**				
<0.25	21 (5.9, 2.0-16.4)	103 (23.8, 15.1-35.4)	20 (4.2, 1.4-11.9)	110 (19.3, 12.2-29.0)
0.25-0.5	145 (33.3, 22.5-46.1)	182 (47.7, 35.9-59.7)	127 (25.0, 16.5-36.0)	261 (46.1, 35.8-56.8)
0.5-1	173 (37.6, 26.3-50.4)	91 (19.5, 11.8-30.5)	233 (45.5, 34.7-56.6)	132 (23.1, 15.3-33.3)
>1	103 (23.3, 13.9-36.3)	51 (9.1, 4.2-18.3)	147 (25.4, 16.9-36.3)	70 (11.5, 6.3-20.3)
**RDT result**				
Negative	416 (95.0, 91.9-97.0)	406 (95.2, 92.5-96.9)	397 (76.1, 71.4-80.2)	499 (86.7, 82.4-90.1)
Positive	26 (5.0, 3.0-8.1)	21 (4.8, 3.1-7.5)	130 (23.9, 19.8-28.6)	74 (13.3, 9.9-17.6)
**Slept under net last night**				
No	217 (47.5, 36.5-58.7)	148 (34.6, 24.8-45.8)	305 (59.7, 50.2-68.6)	46 (8.3, 4.5-14.7)
Yes	225 (52.5, 41.3-63.5)	279 (65.4, 54.2-75.2)	222 (40.3, 31.4-49.8)	527 (91.7, 85.3-95.5)
				

*Weighted percentage (basic probability weights).

CI confidence interval, EVI enhanced vegetation index, HF health facility, PCA principal component analysis, Precip. precipitation, SES socioeconomic status, SMC seasonal malaria chemoprevention, Temp. temperature.

Note: qPCR outcome was not available for all participants and is not included in this table.

### Intervention effect on malaria prevalence and LLIN usage

Weighted baseline *Plasmodium falciparum* infection prevalence by RDT was 5.0% (95% CI: 3.0, 8.1%) in the control group and 4.8% (95% CI: 3.1, 7.5%) in the intervention group, which increased to 23.9% (95% CI: 19.8, 28.6%) in the control group and 13.3% (95% CI: 9.9, 17.6%) in the intervention group at endline. The package of interventions was associated with an adjusted 12.6-percentage point relative reduction in malaria prevalence (RDT) in the intervention group (p = 0.01, 95% CI: -2.7, -22.4) compared to the counterfactual when adjusting for age group, number of people living in the daara, socioeconomic status, distance to nearest health facility, seasonal malaria chemoprevention, and environmental factors including EVI and temperature (Table 2).

**Table 2:** Difference in difference of main outcomes among the talibés.

Indicator	Intervention		Control			
	Baseline n (%*)	Endline n (%*)	Baseline n (%*)	Endline n (%*)	Adjusted RD (95% CI)	Adjusted OR (95% CI)
RDT-derived malaria					-12.6 (-2.7, -22.4) p = 0.01	0.42 (0.16, 1.1) p = 0.07
Yes	21 (4.8%)	74 (13.3%)	26 (5.0%)	130 (23.9%)		
No	406 (95.2%)	499 (86.7%)	416 (95.0%)	397 (76.1%)		
qPCR-derived malaria**					-12.3 (-1.7, -22.8) p = 0.02	0.36 (0.15, 0.86) p = 0.021
Yes	63 (14.7%)	147 (27.8%)	11 (8.8%)	63 (43.1%)		
No	359 (85.3%)	384 (72.2%)	95 (91.2%)	82 (56.9%)		
Prior night net use					42.3 (33.8, 50.7) p < 0.001	12.5 (7.66, 20.5) p < 0.001
Yes	279 (65.4%)	527 (91.7%)	225 (52.5%)	222 (40.3%)		
No	148 (34.6%)	46 (8.3%)	217 (47.5%)	305 (59.7%)		
						

*Weighted percentage.

**Available for a subset of results.

CI confidence interval, OR odds ratio, qPCR quantitative polymerase chain reaction, RD risk difference, RDT rapid diagnostic test.

qPCR results were available for 78.5% of participants (the majority that were not available were from the endline control group). Distribution of characteristics such as age and gender were similar among the population with DBS available and the population missing DBS. Weighted qPCR derived prevalence among this subgroup was 8.8% (95% CI: 3.9, 18.7%) and 14.7% (95% CI: 11.2, 18.9%) among baseline control and intervention groups, respectively, increasing at endline to 43.1% (95% CI: 33.4, 53.3%) among the control group and 27.8% (95% CI: 22.5, 33.8%) among the intervention group. Using prevalence by q-PCR, available only for a subset of results, the package of interventions was associated with an adjusted 12.3-percentage point reduction (p = 0.02, 95% CI: -1.7, -22.8) compared to the counterfactual.

Reported LLIN usage the previous night decreased in the control group from a weighted 52.5% (95% CI: 41.3, 63.5%) to 40.3% (95% CI: 31.4, 49.8%) between baseline and endline and increased in the intervention group from 65.4% (95% CI: 54.2, 75.2%) to 91.7% (95% CI: 85.3, 95.5%). Among the talibés, the package of interventions was associated with an adjusted 44-percentage point increase in reported prior night net use in the intervention group (p < 0.001, 95% CI: 36.3, 51.6) compared to the counterfactual after adjusting for age group, number of people living in the daara, and socio-economic status.

### Intervention effect on passive malaria incidence

Among the general population in the study health facility catchments and controlling for environmental variables, there were 2.9 fewer cases per 1,000 population (95% CI: -4.7, -1.2) in the intervention catchments compared to the counterfactual during the four months following the intervention period (October 2021-January 2022). There were also 3.8 fewer cases per 1,000 population (95% CI: -11.8, 4.1) of malaria among the talibés in the intervention group, however this finding was not statistically significant.

### Feasibility and acceptability of the interventions

Daara-level LLIN coverage increased from 4.4% at baseline to 13.0% at endline in the control group and from 17.4% to 65.2% in the intervention group. Twenty-three of the twenty-six schools (88.5%) in the endline intervention group had a DSDAARA. Of the twenty-one DSDAARA present to be interviewed, 52.4% reported they had tested or treated for malaria since the training; 90% of those who had not tested listed RDT stockouts as the reason. The school masters reported that they and the talibés were very comfortable with the interventions (including being tested by a peer) and the DSDAARA reported they were very comfortable testing and treating for malaria. Malaria knowledge, i.e., identifying mosquitoes as the method of transmission of malaria, fever as a symptom of malaria, and using a bed net as a method of prevention against malaria was high at baseline (over 94% correct across the three indicators) and endline (100% correct) in both arms of the study.

### Gold miners

#### Descriptive analysis.

Overall, the miners were 61.0% male; there was a higher percentage of female miners in the endline control group than in other groups (Table 3). The majority of gold miners were in the age range of 25–49 years (61.0%) and reported having no formal education (65.7%). Country of citizenship differed substantially between endline and baseline; 52.9% of gold miners at baseline but only 28.2% (21.6% in control and 35.3% in intervention) at endline were Senegalese citizens. At endline, 207 miners (36.9%) in the control group and 192 miners (35.6%) in the intervention group reported they were present at baseline.

**Table 3:** Gold miners: participant characteristics, malaria prevalence, and LLIN usage.

	Baseline Control	Baseline Intervention	Endline Control	Endline Intervention
	N = 424 (%, 95% CI)	N = 446 (%, 95% CI)	N = 561 (%, 95% CI)	N = 539 (%, 95% CI)
**Health facility**				
Mamakhono	127 (30.0, 25.8-34.5)	—	165 (29.4, 25.8-33.3)	—
Sambrambougou	297 (70.0, 65.5-74.2)	—	396 (70.6, 66.7-74.2)	—
Diakhaling	—	213 (47.8, 43.2-52.4)	—	244 (45.3, 41.1-49.5)
Khossanto	—	233 (52.2, 47.6-56.8)	—	295 (54.7, 50.5-58.9)
**Age group (years)**				
13-24	120 (28.3, 24.2-32.8)	135 (30.3, 26.2-34.7)	190 (33.9, 30.0-37.9)	189 (35.1, 31.1-39.2)
25-49	285 (67.2, 62.6-71.5)	278 (62.3, 57.7-66.7)	315 (56.1, 52.0-60.2)	323 (59.9, 55.7-63.9)
50 and older	19 (4.5, 2.9-6.9)	33 (7.4, 5.3-10.2)	56 (10.0, 7.8-12.8)	27 (5.0, 3.5-7.2)
**Sex**				
Male	287 (67.7, 63.1-72.0)	292 (65.5, 60.9-69.7)	259 (46.2, 42.1-50.3)	363 (67.3, 63.3-71.2)
Female	137 (32.3, 28.0-36.9)	154 (34.5, 30.3-39.1)	302 (53.8, 49.7-57.9)	176 (32.7, 28.8-36.7)
**Level of education**				
No formal education	260 (61.3, 56.6-65.8)	340 (76.2, 72.1-80.0)	384 (68.4, 64.5-72.2)	310 (57.5, 53.3-61.6)
Primary school	41 (9.7, 7.2-12.9)	40 (9.0, 6.6-12.0)	77 (13.7, 11.1-16.8)	108 (20.0, 16.9-23.6)
Koranic school	103 (24.3, 20.4-28.6)	46 (10.3, 7.8-13.5)	74 (13.2, 10.6-16.3)	78 (14.5, 11.7-17.7)
Secondary or Higher	20 (4.7, 3.1-7.2)	20 (4.5, 2.9-6.8)	26 (4.6, 3.2-6.7)	43 (8.0, 6.0-10.5)
**Citizenship**				
Burkina-Faso	22 (5.2, 3.4-7.8)	52 (11.7, 9.0-15.0)	69 (12.3, 9.8-15.3)	58 (10.8, 8.4-13.7)
Guinea	105 (24.8, 20.9-29.1)	22 (4.9, 3.3-7.4)	175 (31.2, 27.5-35.2)	26 (4.8, 3.3-7.0)
Mali	58 (13.7, 10.7-17.3)	131 (29.4, 25.3-33.8)	171 (30.5, 26.8-34.4)	250 (46.4, 42.2-50.6)
Other	22 (5.2, 3.4-7.8)	8 (1.8, 0.9-3.5)	25 (4.5, 3.0-6.5)	15 (2.8, 1.7-4.6)
Senegal	217 (51.2, 46.4-55.9)	233 (52.2, 47.6-56.8)	121 (21.6, 18.4-25.2)	190 (35.3, 31.3-39.4)
**SES (PCA quartile)**				
0-25	151 (35.6, 31.2-40.3)	174 (39.0, 34.6-43.6)	244 (43.5, 39.4-47.6)	184 (34.1, 30.3-38.2)
25.1-50	59 (13.9, 10.9-17.5)	55 (12.3, 9.6-15.7)	222 (39.6, 35.6-43.7)	200 (37.1, 33.1-41.3)
50.1-75	132 (31.1, 26.9-35.7)	125 (28.0, 24.1-32.4)	0 (0)	0 (0)
75.1-100	82 (19.3, 15.9-23.4)	92 (20.6, 17.1-24.6)	95 (16.9, 14.1-20.3)	155 (28.8, 25.1-32.7)
**Residence sprayed in last year**				
No	392 (92.5, 89.5-94.6)	438 (98.2, 96.5-99.1)	515 (91.8, 89.2-93.8)	454 (84.2, 80.9-87.1)
Yes	32 (7.5, 5.4-10.5)	8 (1.8, 0.9-3.5)	46 (8.2, 6.2-10.8)	85 (15.8, 12.9-19.1)
**Sleeping structure**				
Informal	149 (35.1, 30.7-39.8)	165 (37.0, 32.6-41.6)	375 (66.8, 62.8-70.6)	255 (47.3, 43.1-51.5)
Modern	64 (15.1, 12.0-18.8)	37 (8.3, 6.1-11.2)	40 (7.1, 5.3-9.6)	45 (8.3, 6.3-11.0)
Permanent traditional	211 (49.8, 45.0-54.5)	244 (54.7, 50.1-59.3)	146 (26.0, 22.6-29.8)	239 (44.3, 40.2-48.6)
**Household size, mean (SD, CI)**	4.3 (2.4, 4.0-4.5)	4.4 (3.4, 4.1-4.7)	3.1 (1.4, 3.0-3.2)	3.0 (1.7, 2.8-3.1)
**EVI, mean (SD, CI)**	0.44 (0.07, 0.43-0.44)	0.48 (0.07, 0.47-0.48)	0.28 (0.04, 0.28-0.28)	0.29 (0.03, 0.28-0.29)
**Temp. (C), mean (SD, CI)**	27.5 (0.4, 27.5-27.6)	26.8 (0.9, 26.7-26.8)	36.7 (0.5, 36.6-36.7)	37.1 (0.9, 37.0-37.2)
**Precip. (mm), mean (SD, CI)**	325.1 (4.7, 324.7-325.6)	328.9 (2.7, 328.7-329.2)	0.16 (0.0, 0.16-0.16)	0.13 (0.0, 0.12-0.13)
**Distance to nearest HF (km)**				
<0.5	205 (48.3, 43.6-53.1)	117 (26.2, 22.4-30.5)	75 (13.4, 10.8-16.4)	83 (15.4, 12.6-18.7)
0.5-1.0	14 (3.3, 2.0-5.5)	137 (30.7, 26.6-35.2)	48 (8.6, 6.5-11.2)	237 (44.0, 39.8-48.2)
>1.0	205 (48.3, 43.6-53.1)	192 (43.0, 38.5-47.7)	438 (78.1, 74.5-81.3)	219 (40.6, 36.6-44.8)
**RDT result**				
Negative	290 (68.4, 63.8-72.6)	323 (72.4, 68.1-76.4)	525 (93.6, 91.2-95.3)	505 (93.7, 91.3-95.5)
Positive	134 (31.6, 27.4-36.2)	123 (27.6, 23.6-31.9)	36 (6.4, 4.7-8.8)	34 (6.3, 4.5-8.7)
qPCR result*				
Negative	133 (31.4, 27.1-35.9)	165 (37.2, 32.9-41.8)	311 (55.4, 51.3-59.5)	314 (58.3, 54.0-62.4)
Positive	291 (68.6, 64.1-72.9)	278 (62.8, 58.2-67.1)	250 (44.6, 40.5-48.7)	225 (41.7, 37.6-46.0)
**Slept under net last night**				
No	163 (38.4, 33.9-43.2)	183 (41.0, 36.6-45.7)	259 (46.2, 42.1-50.3)	306 (56.8, 52.5-60.9)
Yes	261 (61.6, 56.8-66.1)	263 (59.0, 54.3-63.4)	302 (53.8, 49.7-57.9)	233 (43.2, 39.1-47.5)
				

*Three individuals in the baseline intervention group were missing qPCR results.

CI confidence interval, EVI enhanced vegetation index, HF health facility, PCA principal component analysis, Precip. precipitation, SES socioeconomic status, Temp. temperature.

### Intervention effect on malaria prevalence and LLIN usage

Baseline malaria prevalence by RDT was 31.6% (95% CI: 27.4, 36.2%) in the control group and 27.6% (95% CI: 23.6, 31.9%) in the intervention group. At endline, prevalence was 6% in both groups (95% CI: 4.7, 8.8% in the control group; 4.5, 8.7% in the intervention group). Among the gold miners, there was no measured association between the package of interventions and malaria prevalence by RDT (aRD: 1.2-percentage point decrease in prevalence in the intervention group compared to the counterfactual, p = 0.66, 95% CI: -6.7, 4.3) after adjusting for age group, gender, citizenship, socioeconomic status, education level, number of people living at the mine, distance to nearest health facility, indoor residual spraying, type of sleeping structure, and temperature (Table 4). In the sensitivity analysis, the analysis was restricted to individuals who reported participating at both the baseline and endline surveys, and the results were similar (aRD: 2.4 percentage-point decrease in prevalence, p = 0.60, 95% CI: -11.1, 6.3).

**Table 4:** Difference in difference of main outcomes among the gold miners.

Outcome	Intervention		Control			
	Baseline n (%)	Endline n (%)	Baseline n (%)	Endline n (%)	Adjusted RD (95% CI)	Adjusted OR (95% CI)
RDT-derived malaria					-1.2 (-6.7, 4.3) p = 0.66	0.84 (0.42, 1.65) p = 0.62
Yes	123 (27.6%)	34 (6.3%)	134 (31.6%)	36 (6.4%)		
No	323 (72.4%)	505 (93.7%)	290 (68.4%)	525 (93.6%)		
qPCR-derived malaria*					-1.7 (-12.6, 9.1) p = 0.75	0.94 (0.50,1.75) p = 0.84
Yes	278 (62.8%)	225 (41.7%)	291 (68.6%)	250 (44.6%)		
No	165 (37.3%)	314 (58.3%)	133 (31.4%)	311 (55.4%)		
Prior night net use					-5.0 (-33.2, 23.2) p = 0.73	0.80 (0.22, 2.89) p = 0.73
Yes	263 (59.0%)	233 (43.2%)	261 (61.6%)	302 (53.8%)		
No	183 (41.0%)	306 (56.8%)	163 (38.4%)	259 (46.2%)		
						

*Three participants were missing qPCR results.

CI confidence interval, OR odds ratio, RD risk difference, RDT rapid diagnostic test, qPCR quantitative polymerase chain reaction.

qPCR results were available for 99.8% of gold miners; prevalence by qPCR also decreased between baseline and endline and was much higher in all groups than prevalence by RDT. Using this outcome the package of interventions was not associated with a difference in malaria prevalence in the intervention group compared to the counterfactual (aRD: -1.7; p = 0.75, 95% CI: -12.6, 9.1) (Table 4). Among individuals who reported participating at baseline and endline surveys, the results were similar (aRD: -0.5; p = 0.96, 95% CI: -17.4, 16.5).

Reported LLIN usage decreased between baseline and endline in both groups. In the control group, it decreased from 61.6% (95% CI: 56.7, 66.2%) to 53.8% (95% CI: 49.6, 58.0%) and in the intervention group from 59.0% (95% CI: 54.2, 63.6%) to 43.2% (95% CI: 39.0, 47.5%). There was no measured association between the package of interventions and reported prior night net use compared to the counterfactual (aRD: 5.0; p = 0.73, 95% CI: -33.2, 23.2), after adjusting for age group, gender, citizenship, education level, socioeconomic status, distance to the nearest health facility, type of sleeping structure, number of people living at the mine, and indoor residual spraying. Among individuals who reported participating in both surveys, the results were similar (aRD: -1.5; p = 0.94, 95% CI: -39.7, 36.8).

### Intervention effect on passive malaria incidence

Among the general population in the study health facility catchments and controlling for environmental variables, there were 31.3 fewer cases per 1,000 population (95% CI: -59.6, -3.0) in the intervention catchments compared to the counterfactual during the four months following the intervention period (October 2021-January 2022). There were also 10.5 fewer cases per 1,000 population (95% CI: -25.0, 4.0) of gold miners in the intervention group compared to the counterfactual, but this finding was not statistically significant.

### Feasibility and acceptability of the interventions

Reported LLIN coverage among the gold miners increased on average between baseline and endline from 64.4% to 68.6% in the control group and from 62.8% to 73.1% in the intervention group. At endline, 452 gold miners (97.6%) reported that a peer from their site had been trained in the diagnosis and treatment of malaria and 281 (52.1%) reported they had been offered testing and treatment for malaria since the beginning of the study. All respondents said they felt comfortable being provided a mosquito net for malaria prevention and tested for malaria by a peer; however, 65.8% reported that distribution of nets at a health facility would have made this intervention more acceptable. Only 13.0% of the gold miners in the endline intervention group said the first place they would go to get tested for malaria would be to the DSDOR.

All eight DSDORs reported they had tested and treated for malaria since the training with an average number of 8 (range 4–15) tests reported. All reported high satisfaction with training and comfort in performing testing and treating for malaria. DSDORs reported travel difficulties to get to the site, gold miner refusal, mobility of the miners, and ACT stockouts as challenges. 100% of the DSDORs reported they carried out PECADOM+ for those with fever every week; challenges to conducting PECADOM+ were fear, refusal, or unavailability of gold miners, not having enough tools/forms, and lack of transportation.

At baseline, over 99% of gold miners identified mosquitoes as the mode of transmission for malaria, listed a correct prevention method, and identified fever as a symptom of malaria. At endline, knowledge of the mode of transmission and prevention remained at over 99% and knowledge of fever as a symptom of malaria decreased slightly in both groups.

## Discussion

We found that among talibés, a targeted package of interventions was associated with an adjusted 13-percentage point reduction in malaria prevalence and an adjusted 44-percentage point increase in reported net usage. Among gold miners, the package of interventions was not associated with a difference in malaria prevalence or reported net usage in the intervention group compared to the counterfactual. The dissimilarity in effectiveness of the intervention package among the accessible student populations at urban boarding schools compared to the highly mobile, diverse, and hard-to-reach gold mining populations in remote regions highlights the need for individualized approaches for different malaria high-risk populations.

Delivering routine interventions in a targeted package can be an effective approach to reaching talibés. Previous studies in Senegal and other countries have called for increased attention on interventions for school-age children and adolescents; despite an increased risk of malaria, these populations are often overlooked [6–9,29]. A new approach was used for LLIN distribution in this study: delivery via peer community health workers. Targeted LLIN delivery as part of the package was associated with an increase in reported net usage and reduction in malaria prevalence in talibés. Usage increased between baseline and endline despite higher temperatures at endline, which can discourage net usage in general [30]. Previous research in Senegal has identified that non-use of LLINs is associated with increased odds of malaria among children and adolescents [6]. This result is consistent with findings in other countries such as Kenya and Rwanda; ownership and usage of LLINs was associated with lower odds of malaria among these age groups [31,32]. Challenges remain to ensure adequate LLIN coverage in daaras: in formative research that preceded this study, respondents indicated the daaras often have insufficient quantities of nets, standard nets can be too small to adequately cover the congregate sleeping areas, and older students may get priority over younger students when there are not enough nets [5].

This study also provides evidence that increased case management via a peer community health worker in the daara was part of an effective package of interventions for talibés despite reported challenges such as RDT stockouts. Both the talibés and the DSDAARAs reported high acceptability in receiving and delivering testing and treatment among peers.

Other studies have found that schools are a good place to deliver health interventions and that effective diagnosis and treatment of malaria can be improved via provision of case management at schools [5,6]. Additionally, while this study found high levels of malaria knowledge among the school masters, malaria knowledge among individual talibés was not assessed. Other studies in Senegal have found low levels of malaria knowledge among children and adolescents and that education in tandem with interventions such as LLIN distribution is an effective approach for increasing LLIN use [2,5].

Among the gold miners, we did not find evidence that the package of interventions was effective in reducing malaria prevalence, malaria incidence, or increasing reported net usage in this population with high rates of turnover. It is possible that higher coverage of the DSDOR testing or LLIN interventions could have led to an impact. DSDOR and miners reported high levels of acceptability of the testing intervention; however, only 52.1% of gold miners in the intervention group at endline said they were offered testing and treatment for malaria, and most only once, and the testing rates reported by DSDORs were very low. We did find some evidence of reduced incidence of malaria among the general population in the intervention health facility catchments compared to the counterfactual. However, this indicator is difficult to interpret because we did not control for other covariates such as LLIN and IRS coverage and the potential impact of the intervention in the whole population is diluted, making it challenging to determine causality.

It is commonly accepted that gold miners are a group that is difficult to study [10]. Mobility and turnover of the miners was a big challenge in this study: at endline, 36.2% of gold miners reported that they were present at baseline, and the largest demographic majority of miners shifted from Senegalese citizens at baseline to Malian at endline. DSDORs placed at the gold mines also reported that the mobility of the miners was a great challenge to acting as a CHW. The mobility patterns of the miners are diverse; for example, formative research found that the influx of gold miners can occur throughout the year, in particular if a new gold vein is discovered, and movement patterns differ between groups of different nationalities [5]. Populations that are highly mobile have been found to have more health problems, less access to care, and lower net usage than settled populations [33,34]. Formative research among the gold miners in Senegal also found that the use of LLINs is not routine [5].

It is possible the intervention impact was inequitable amongst the target population and may have failed to reach those who are marginalized or otherwise experiencing the most barriers and risk. Stigma or cultural differences among the miners can be a barrier to interventions; for example, there is mistrust amongst the different gold mining populations, in particular between those who are from Senegal and those who are not, and some communities do not want to integrate with the others [35]. Therefore, it is possible that DSDORs were more likely or able to provide services to some gold miners and not others, which may have led to low utilization of the community health worker at the mine. Travel difficulties in getting to the mine was the most common challenge to testing and treating reported by DSDORs, indicating a major logistical barrier in access to the DSDOR across all the gold miners in the study. Additionally, gold miners in Senegal face numerous risks in the industry of mining; malaria is one of many occupational hazards and they may be more concerned with where they will sleep, what they will eat, and other risks than with worrying about malaria prevention or treatment [5,10].

There were a few limitations of this study. A difference-in-difference analysis requires the parallel trend assumption, which asserts that in the absence of the intervention, the difference between the intervention and control groups is constant over the time period; only two time points does not allow for testing if the assumption holds true [36]. However, randomization and the short time period between the two surveys support the assumption [37,38]. Evaluating the intervention after a short time period was necessary due to the short transmission season but also has some limitations: it may be easier to find an impact after only 4–5 months than after a longer period, and more follow up would be needed to gauge the sustainability of the intervention and how often the nets need to be redistributed and peer CHWs need to be trained. A small number of randomized units is also a limitation: there were two health facility catchments per arm for each population group. Additionally, among the talibés, only a subset of PCR results was available due to the loss of a package of samples in the field. In the incidence analysis, the data were not available to control for LLIN and IRS coverage, among other potential confounding variables, which could bias the findings. Some of the questions that were asked of the school masters (e.g., questions on malaria knowledge) likely do not reflect knowledge among the individual talibés themselves. The students and the DSDAARA may have also felt pressured to answer positively about their experience while interviewed nearby their peers and school masters; this social desirability bias could bias the results away from the null. Among the gold miners, extensive turnover at the mines was a limitation; however, in the sensitivity analysis aimed at assessing the influence of turnover, the findings remained similar across all outcomes to those of the full cohort of participants.

While we did control for environmental variables in the models, it is possible that there are other aspects of seasonality that were not controlled for and may have influenced the results. For example, while the trend among the gold miners of higher prevalence at baseline than endline was consistent with trends in seasonal malaria incidence in the study districts during the same period (via routine surveillance data), the trend of lower prevalence at baseline than endline among the talibés was not consistent with trends in seasonal malaria incidence in the study districts during the same period [5]. This could be due to other aspects of seasonality that differ between Kaolack and Saraya, or unaccounted for behavior such as travel over or differences in access to care during and after the winter holidays.

## Conclusions

The findings of this study provide evidence that a package of interventions including targeted LLIN delivery, peer community case management, and malaria education was associated with an increase in reported net usage and reduction in malaria prevalence among talibés in Kaolack District, Senegal. For this high-risk population, routine interventions delivered in a more targeted manner had beneficial effects on malaria indicators and were feasible; this is highly needed as adolescents and school age children are a group that are often overlooked compared to other groups such as pregnant women and children under five. This package of interventions can be utilized in daaras throughout Senegal and other countries with boarding school populations.

The effectiveness of this package of interventions was not found among gold miners in Saraya district, working in remote, rural, gold mines. While talibés spend long periods of time at daaras and live in a hierarchy with school masters who are responsible for them, miners are truly a “hard to reach” population: they are mobile, many are from other countries, heterogeneous in demographics, not organized under a structure within which to target interventions, they are often vulnerable and may not know how to access health prevention. Future research among gold miners will require advanced participatory methods to understand which interventions and delivery methods will most benefit this population: delivering routine interventions in this highly targeted manner was insufficient.
